# Estimation of a whole plant Q10 to assess seagrass productivity during temperature shifts

**DOI:** 10.1038/s41598-019-49184-z

**Published:** 2019-09-02

**Authors:** Lina M. Rasmusson, Martin Gullström, Pontus C. B. Gunnarsson, Rushingisha George, Mats Björk

**Affiliations:** 1 Seagrass Ecology & Physiology Research Group, Department of Ecology, Environment and Plant Sciences, Stockholm University, Stockholm, Sweden; 2 Regional Climate Group, Department of Earth Sciences, University of Gothenburg, Gothenburg, Sweden; 3 Department of Biological and Environmental Sciences, University of Gothenburg, Kristineberg, Fiskebäckskil Sweden; 4 Tanzania Fisheries Research Institute (TAFIRI), Dar es Salaam, Tanzania

**Keywords:** Ecosystem ecology, Photosynthesis

## Abstract

Through respiration and photosynthesis, seagrass meadows contribute greatly to carbon and oxygen fluxes in shallow coastal waters. There is increasing concern about how shallow-water primary producers will react to a near-future climate scenario with increased temperature variation. When modelling primary productivity under high temperature variability, Q10 values are commonly used to predict rate changes depending on biophysical factors. Q10 values are often assumed to be constant and around 2.0 (i.e. a doubling of the rate with a temperature increase of 10 °C). We aimed to establish how the gas exchange of seagrass (*Zostera marina*) tissues at various maturity stages would respond over a broad range of temperatures. Seagrass shoot maturity stage clearly affected respiration and apparent photosynthesis, and the Q10 results indicated a skewed balance between the two processes, with a higher photosynthetic Q10 during periods of elevated temperatures. When estimating whole-plant Q10 in a realistic maximal temperature range, we found that the overall response of a seagrass plant’s net O_2_ exchange balance can be as much as three to four times higher than under ambient temperatures. Our findings indicate that plant tissue age and temperature should be considered when assessing and modelling carbon and oxygen fluctuations in vegetated coastal areas.

## Introduction

Seagrasses are submerged marine angiosperms colonising areas of soft-bottom sediment with an extensive network of below-ground tissues (i.e. roots and rhizomes). Below-ground tissues are important for nutrient uptake, clonal reproduction, and anchorage^1,2^. However, to support a large build-up of non-photosynthetic underground tissues, high photosynthetic efficiency of above-ground tissues is essential^2^. Seagrass plants fix large amounts of carbon dioxide, i.e. 394–449 gC m^−2^ year^−1^ globally^3^, while a portion of the fixed carbon is released back into the water through respiration. This portion can be large, although quite variable, due to the large amount of below-ground seagrass tissue^2^. High seagrass productivity can therefore greatly influence coastal carbon and oxygen fluxes, and subsequently pH^4–7^. Both photosynthesis and respiration are strongly influenced by abiotic factors such as water temperature that normally cause increased rates up to an optimal level beyond which rates drop^8–11^. Time of day is important because there is substantial diel variation in rates^12–14^. The availability of oxygen and carbon also varies during the day as a result of community respiration, influencing both photosynthesis and respiration, which are also influenced by light levels and biotic factors such as the age of leaf tissue^15–19^. To make accurate predictions of plant carbon budgets in a changing environment, more detailed knowledge of the variability of respiration and photosynthesis is critical. Measuring productivity of different parts of the seagrass plant and in tissues of various ages could help make plant carbon budgets more accurate, as information regarding within-plant variability is currently sparse. It is well known that photosynthetic rates differ with the maturity of leaf tissue^16–19^; however, study of the respiration of seagrasses with regard to tissue age of individual plants has received insufficient attention. Nonetheless, from the whole-meadow perspective, community respiration rates have been shown to increase with age as more respiring biomass is present and microbial activity in the sediment is enhanced by the cumulative input of plant material^20^. Hence, a shift in the metabolic trophic state of seagrass meadows can be detected with age^21^, supporting the notion of age-dependent productivity in seagrass systems. Q10, a coefficient often used to predict the rate of metabolic change given a specific temperature increase, is commonly used to determine productivity responses in terrestrial and marine ecosystems^22,23^, including in seagrass meadows^8,10,24,25^. A Q10 value of 2 (i.e. a doubling of the metabolic rate with a temperature increase of 10 °C) is often used in climate models to predict the temperature response of a plant or ecosystem^26–29^. However, variations in the Q10 ratio depend on the initial temperature (used for calculating Q10) and/or the ranges of temperatures chosen^23,30–32^, so the general assumption of a doubling of the metabolic rate can be misleading.

**Figure 1:**
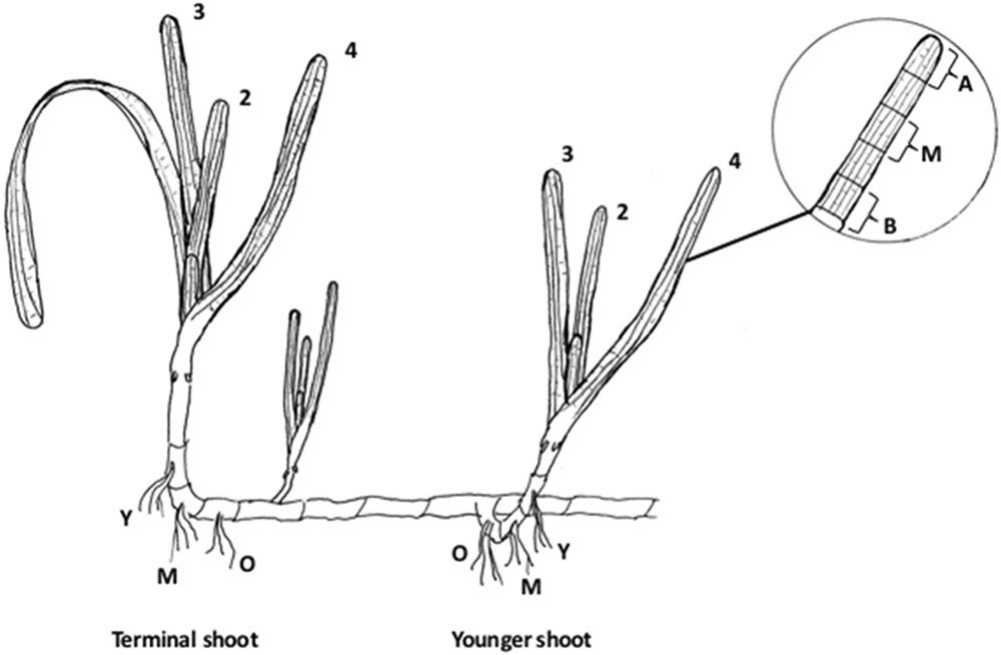
Genet of *Zostera marina* showing the older terminal shoot and the second youngest shoot examined. For each ramet, the rates of respiration and apparent photosynthesis were recorded for leaves 2, 3, and 4. Each leaf was divided into three parts, i.e. basal (B), mid (M), and apex (A), as shown in the inset. The below-ground respiration was measured in younger (Y), middle-aged (M), and older (O) segments of the rhizome belonging to each shoot (illustration by LMR).

Seagrasses can reproduce sexually, but rely largely on vegetative propagation by clonal growth^1,33^. Asexual clonal growth is accomplished by extension and branching of rhizomes where new shoots, called ramets, form. Clonal expansion of the temperate seagrass *Zostera marina* L. occurs when a terminal shoot, often dormant over winter, extends and spreads horizontally through the sediment. Younger shoots emerge from side branches, with the youngest shoot positioned slightly behind the leading shoot^33,34^ (Fig. 1).

To understand the variability of plant productivity on an individual ramet basis, we assessed how the respiratory and photosynthetic rates of the temperate seagrass *Z*. *marina* vary relative to tissue age under ambient temperatures and during higher temperature events. The temperature coefficient Q10 was used to estimate: (1) how photosynthetic and respiratory rates will change with a temperature increase of 10 °C; and (2) how plant productivity will respond to various temperature intervals. We hypothesised that: (a) the respiration rate will be higher in younger seagrass tissues (i.e. both above- and below-ground parts) in response to potentially higher energy demand; (b) the basal parts of leaves, where meristematic growth occurs, will have higher respiration rates than more mature leaf parts; and (c) photosynthetic rates will be higher in the leaf apex and middle part than in the leaf base due to a potentially greater abundance of light-harvesting chloroplasts.

## Results

**Figure 2:**
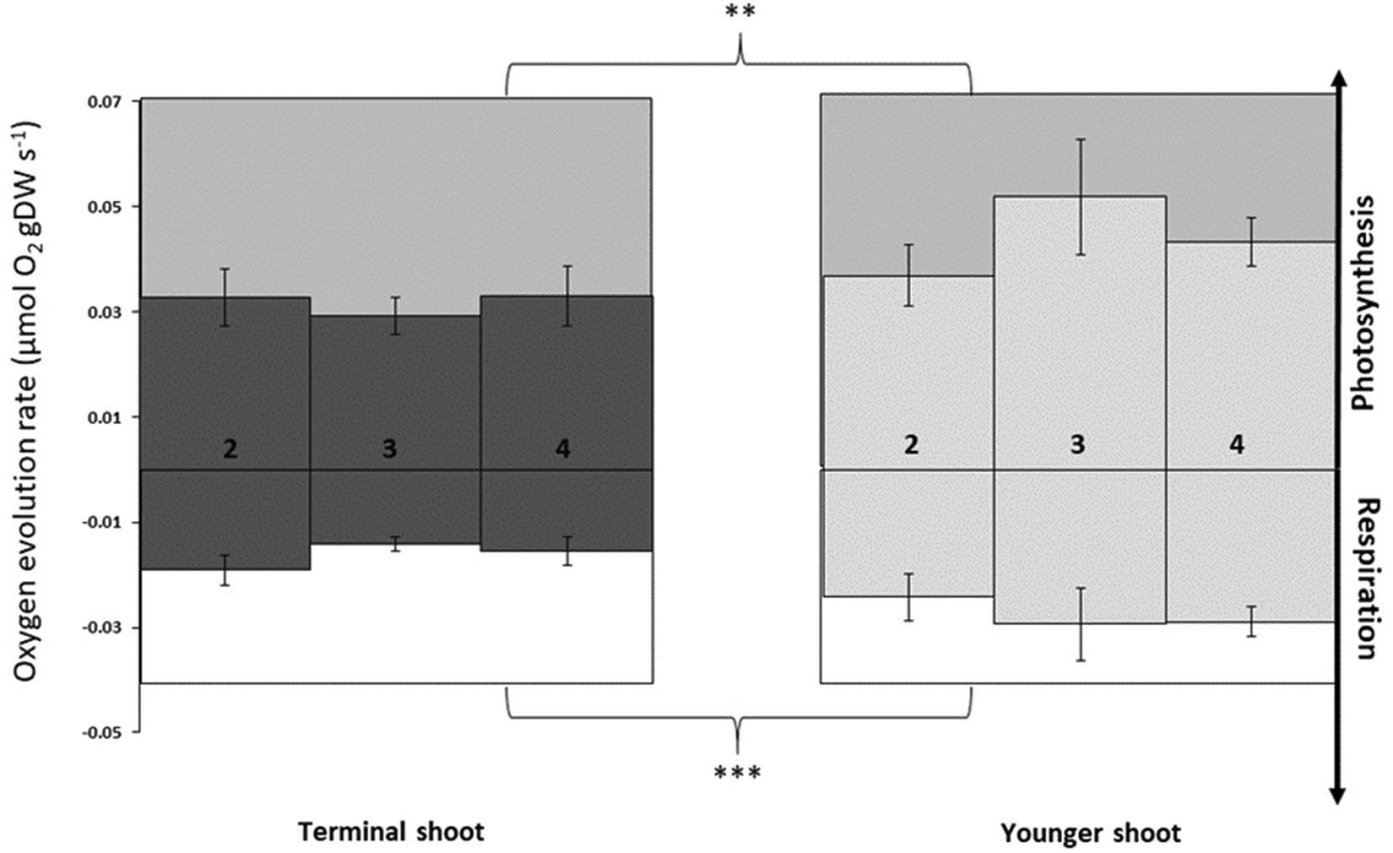
Apparent photosynthesis and respiratory rates for *Zostera marina* of different maturation stages. Mean (±SE) apparent photosynthetic and respiratory rates for leaves (leaf rank indicated on the *x*-axis) and shoots of different maturation stages. No differences were detected in leaf rank, whereas differences in shoot age are indicated by the shaded boxes (*n* = 33–35). **p < 0.01, ***p < 0.001.

**Figure 3:**
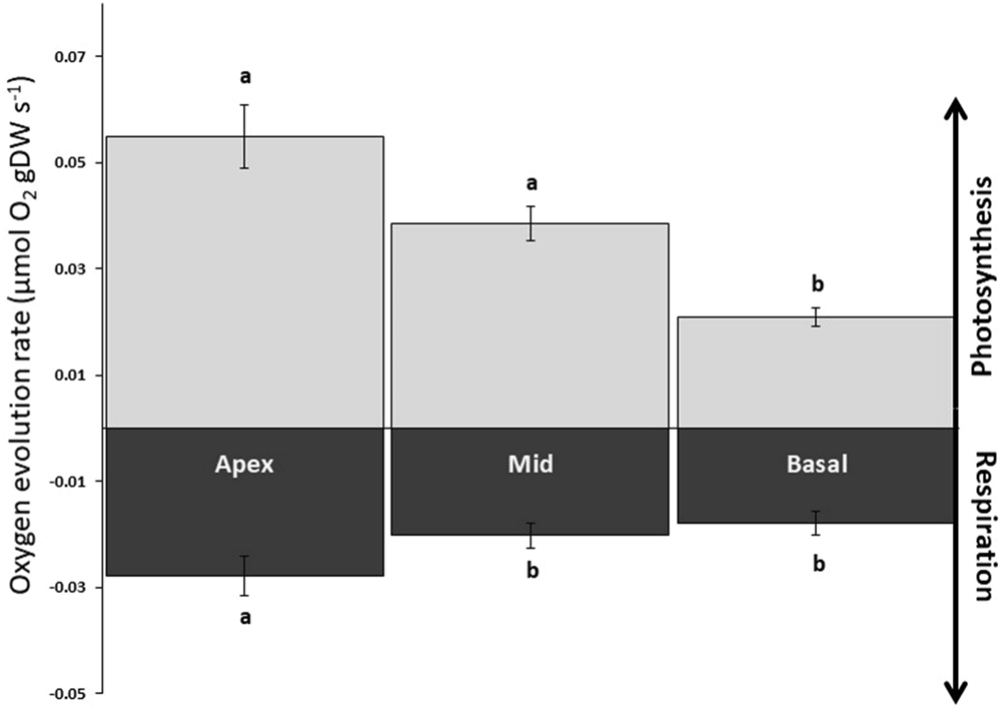
Apparent photosynthetic and respiratory rates of different leaf parts in *Zostera marina*. Mean (±SE) rates for different parts of seagrass leaves, with shoots and leaf ranks pooled (*n* = 15). Letters “a” and “b” above the bars indicate significant differences among leaf parts, *p* < 0.01.

**Figure 4:**
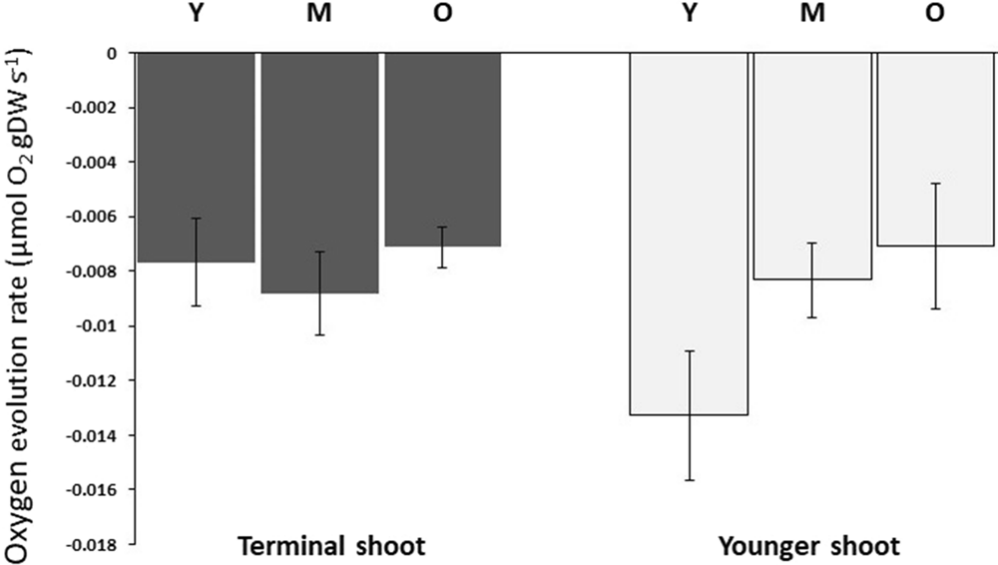
Below-ground tissue respiration in *Zostera marina*. Mean (±SE) respiration of below-ground tissues separated into younger (Y), middle-aged (M), and older (O) rhizome segments with attached roots belonging to either the terminal or younger shoot at ambient temperature (*n* = 4–6). No significant age-related differences in below-ground respiration were found.

Dark respiration rates were higher in younger shoots than older terminal shoots (Fig. 2, Table 1, Tukey’s test, *p* < 0.001). However, respiration rate did not differ among individual leaf ranks within a shoot (Fig. 2, Table 1). Within leaves, apex sections had higher respiration rates than did mid and basal sections (Fig. 3, Table 1, Tukey’s test, *p* < 0.01). Photosynthetic rates were significantly higher in younger shoots than older ones (Fig. 2, Table 1, Tukey’s test, *p* < 0.01), but were not affected by leaf rank (Fig. 2, Table 1). Within leaves, photosynthetic rates differed among sections, with highest rates in apex sections, followed by the mid-sections and, finally, the basal sections (Fig. 3, Table 1, Tukey’s test, *p* < 0.01). No interaction between shoot age and leaf rank, leaf part and leaf rank, or leaf part and shoot age was found for either respiration or photosynthetic rate (Table 1). In below-ground parts, there were no differences in respiration related to either shoot age or tissue age (Fig. 4).

**Table 1:** Summary of three-factor ANOVAs for dark respiration and apparent photosynthetic rates of *Zostera marina* shoots and leaves.

Source of variation	df	Dark respiration			Apparent photosynthesis		
		MS	*F*	p	MS	*F*	p
Shoot	1	0.001	24.3	***	0.003	10.8	**
Leaf	2	0.000	0.10	0.922	0.000	0.23	0.794
Part of leaf	2	0.000	5.80	**	0.005	17.6	***
Shoot × Leaf	2	0.000	1.90	0.157	0.000	0.42	0.656
Shoot × Part of leaf	2	0.000	0.70	0.488	0.000	0.25	0.776
Leaf × Part of leaf	4	0.000	1.50	0.196	0.000	0.23	0.920
Shoot × Leaf × Part of leaf	4	0.000	0.30	0.909	0.000	0.38	0.820
Residual	160	0.000			0.000		

**p < 0.01, ***p < 0.001.

**Figure 5:**
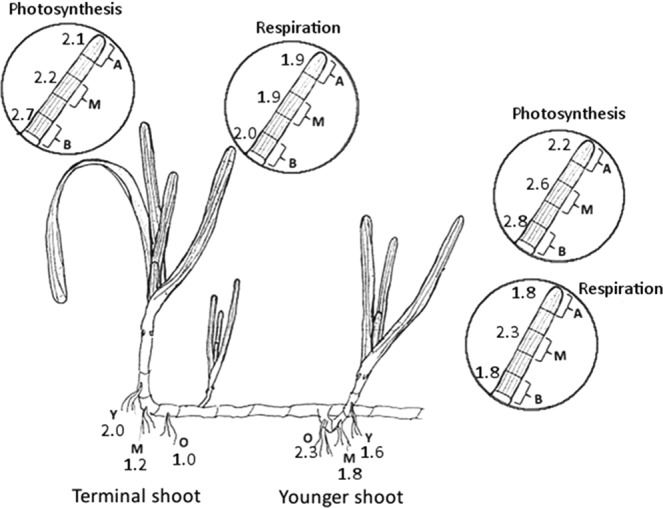
Respiratory and photosynthetic Q10 values in *Zostera marina*. Determined for the 19–29 °C interval for different parts of seagrass leaves (pooled within leaf rank) and for below-ground structures belonging to the older terminal shoot and the younger shoot. Average Q10 values when combining all tissue types were 2.4 for photosynthesis, 1.9 for above-ground respiration, and 1.7 for below-ground respiration (*n* = 5) (illustration by LMR).

Dark respiration and photosynthetic rates for both leaves and below-ground tissues were consistently higher at elevated temperatures (as reflected in the Q10 values presented in Fig. 5). In all tissue types, average Q10 values in the 19–29 °C interval for photosynthesis, above-ground respiration, and below-ground respiration were 2.4, 1.9, and 1.7, respectively, when all tissues were pooled. Q10 was calculated for the different leaf and rhizome parts, and the gas exchange results for all leaf parts within a shoot were pooled (Fig. 5). The highest photosynthetic Q10 was found in the basal parts of younger and terminal shoots (Q10 = 2.8 and 2.7, respectively). Q10 values for respiration were highest in the mid-parts of younger shoots (Q10 = 2.3), while the rest of the values were 1.8–2.0. The Q10 of below-ground tissue was highly variable, ranging from 1.0 to 2.3 (Fig. 5).

In calculating whole-plant net O_2_ balance (Eqs (3–5)), a general Q10 of 3.9 was obtained when all shoot and tissue ages were pooled in the calculations. This Q10 was higher, i.e. 4.2, when no change in below-ground temperature was assumed. When calculations were conducted separately, terminal shoots had a lower whole-plant Q10 of 3.7, versus 4.0 for the younger shoots (Table 2).

**Table 2:** Estimated Q10 values for the whole plant’s total O_2_ exchange calculated for two scenarios: with uniform temperature increase in both water and sediment, and with temperature increase only in the water column.

Whole-plant Q10	Temp. change in water and sediment	Temp. change in water only
Terminal shoot	3.7	
Young shoot	4.0	
Average	3.9	4.2

Q10 values vary depending on initial temperature and width of the interval used (Table 3). The respiratory values were generally lower at high temperatures. The highest respiratory Q10 was calculated in the 10–15 °C temperature range, while the lowest Q10 was calculated in the 35–40 °C range. Photosynthetic Q10 values were more variable than respiratory values, with calculated values as low as 0.5 at higher temperatures (i.e. 30–35 °C). No results are presented for photosynthesis at 40 °C, as rates were close to zero at this temperature.

**Table 3:** Q10 values for respiration and apparent photosynthesis in *Zostera marina* calculated for various intervals and initial temperatures (*n* = 17–35).

Temperature range (°C)	Respiratory Q10	App. photosynthetic Q10
10–15	3.8	3.7
10–20	2.8	2.1
10–25	2.7	2.4
10–30	2.3	2.2
10–35	2.2	1.6
10–40	1.0	N.A
15–20	2.1	1.2
15–25	2.3	1.9
15–30	2.0	1.8
15–35	1.9	1.3
15–40	1.7	N.A
20–25	2.4	2.9
20–30	1.9	2.2
20–35	1.8	1.4
20–40	1.6	N.A
25–30	1.5	1.7
25–35	1.6	0.9
25–40	1.4	N.A
30–35	1.7	0.5
30–40	1.4	N.A
35–40	1.2	N.A

## Discussion

With increasing temperature, significant differences in both photosynthetic and respiratory rates were observed among shoots of different ages and among different parts of leaves, while there were no differences among different leaf ranks. Q10 values also differed depending on both plant tissue age and shoot age, and were higher and more variable for photosynthesis than for respiration. Moreover, Q10 values changed based on the temperature range used in the calculations, generally decreasing with increasing temperature.

As hypothesised, photosynthetic rates were significantly higher in the apex and mid-parts than in the younger basal parts of leaves, where the maturity and abundance of chloroplasts were expected to be lower^35^. This has previously been reported in the Mediterranean seagrass species *Posidonia oceanica*, where photosynthetic rates in apex and mid-parts were more than six and five times higher, respectively, than in basal parts^17^. Contrary to our hypothesis, the same pattern was observed for respiration, with the lowest respiration rates in the basal parts of the leaves, where actively dividing meristematic tissue is located^36^ and the energy demand would supposedly be great. This result may relate to the high photosynthesis in the apex, providing both the substrate and need for higher respiration, as maintenance and growth respiration can be strongly dependent on newly produced carbohydrates^37^ and high activity in growing parts requires higher respiration^38^. By comparing respiration between shoots of different ages, we found that young shoots had about 40% higher respiration than did older terminal shoots, reflecting higher metabolic demands of the younger ramets. Our findings coincide with previous findings of higher respiratory rates in younger tissues of *Z*. *marina*^15^ and *Posidonia oceanica*^17^. When comparing different sections of intact leaves using PAM fluorometry, previous studies have detected no differences in electron transport rates or the effective quantum yield of photosynthesis^18,19,39^. However, when leaves had lesions, the apex part had a lower photosynthetic yield^40^. In addition, a lower maximum capacity (*F*_v_/*F*_m_) of the photosynthetic apparatus was detected in the apex parts over the diurnal cycle in the tropical seagrass species *Thalassia testudinum*^18^. Even though PAM fluorometry is a powerful tool for estimating photosynthetic efficiency^40,41^, results can be difficult to compare with gas balance measurements because chlorophyll fluorometry does not capture the impact of mitochondrial respiration or photorespiration on photosynthetic oxygen evolution as do gas exchange measurements. The present results might have been caused by changed activity in any of these processes, and therefore might not necessarily contradict chlorophyll fluorescence measurements.

Our results indicate that leaf respiration and photosynthetic rates were higher in younger shoots and therefore dependent on shoot age. Thus, as younger ramets seem to have higher potential productivity than do older ones, as also reflected in the higher whole-plant Q10, the maturation state and age of the seagrass plants could be important factors to consider when conducting future productivity studies. To capture the productivity of a seagrass meadow as a whole, these results suggest that various ages of shoots and leaves should be considered to obtain a more accurate picture. However, when comparing the respiration or photosynthesis of plants exposed to different environmental conditions or experimental treatments, it is crucial that shoots and/or tissues of similar characteristics and ages be used to minimise the effects of within-plant variation, something also stressed by others^24,39^. In the below-ground tissues, respiration did not display any age-dependent response, so age does not seem to be as important as in above-ground tissues in this species.

As expected, photosynthetic and respiratory rates were consistently higher at the elevated temperature (29 °C), regardless of tissue age, in both above- and below-ground tissues. Overall, in the 19–29 °C temperate range, average Q10 values of all parts of above-ground tissues were 1.9 for respiration and 2.4 for photosynthesis, meaning that respiration would almost double and photosynthesis more than double with a temperature increase of 10 °C. This corresponds to the results observed here in the 20–30 °C degree range, where the average Q10 values were 1.9 and 2.2 for respiration and photosynthesis, respectively. The average respiratory Q10 for roots and rhizomes, i.e. 1.7, was slightly lower than that of the above-ground tissue; nevertheless, the Q10 variability in the below-ground parts displayed no distinct pattern related to age. Considering the Q10 values, higher respiratory oxygen demand from below- and above-ground tissues at elevated temperatures can be assumed to be met by a more pronounced increase in photosynthesis. However, even though the differences in average Q10 values confirm earlier suggestions that the balance between photosynthesis and respiration in *Z*. *marina* is altered during temperature changes^8,24^, our results contradict what was found by these authors, as their Q10 values for *Z*. *marina* were lower for photosynthesis than for respiration. However, these earlier studies used either the second leaf rank^24^ or the mid-section of the leaf^8^, whereas we present a mean value of all leaf ranks and parts. This could influence the results, as we noted that Q10 values differed between types of tissues. Moreover, our calculations were based on apparent photosynthesis, possibly resulting in a higher Q10 value than when net photosynthesis is used^8,24^, as the respiration is subtracted. By calculating Q10 for a wide range of temperatures, we found that in the 20–25 °C range, i.e. the maximum water temperatures normally encountered in shallow seagrass meadows on the Swedish west coast in summertime^5^, the photosynthetic Q10 was 2.9 and respiratory Q10 2.4. As Q10 values for photosynthesis and respiration were quite distinct, we might encounter skewed carbon budgets in an elevated temperature scenario in temperate seagrass meadows with a higher net C gain. Overall, the respiratory Q10 values displayed less among-tissue variation than did the photosynthetic Q10 values, which varied greatly and usually with higher values in younger shoots and leaves than in older ones and were consistently higher in the basal parts than in mid- and apex sections. As we found that the photosynthetic temperature response varied substantially depending on tissue age, the characteristics of the plant material used (i.e. the part or age of the plant considered) could affect comparative studies of Q10 values. In terrestrial plants, the Q10 value has been shown to differ with, for example, the temperature range and the initial temperature used. Measurements at lower temperatures usually give a steeper response curve and thus a higher Q10 value due to more pronounced sensitivity to temperature change^23,30–32,42^. This was also clearly demonstrated in our study, with the highest Q10 response at the lower temperatures, and with the lowest Q10 at 30–35 °C and 35–40 °C for photosynthesis and respiration, respectively. These low Q10 values were likely due to a decline in photosynthetic activity occurring above 30 °C (Rasmusson *et al*. submitted manuscript). Furthermore, the range of temperature intervals also influenced Q10, with most values close to the generic 2.0 value at moderate temperatures (i.e. 15–30 °C) when an interval of 10 °C was used. The underlying temperature conditions therefore determine the Q10 level, and merit consideration when assessing Q10 values in the literature, as these might vary considerably.

When combining the Q10 values for above- and below-ground tissues (assuming an average distribution of biomass between the different tissue types and a below-/above-ground biomass ratio of 2:3, recalculated from^43,44^) to estimate the net oxygen balance at the whole-plant level (also possibly applicable to carbon dioxide), the general Q10 for all tissue and shoot ages was 3.8; however, here too there was a difference depending on shoot age, with the younger shoots resulting in a whole-plant Q10 of 4.0, whereas the rates of terminal shoots resulted in a Q10 of 3.7. This assumes equal temperature changes in the water and sediment; if, however, the temperature change is rapid and the temperature spike only affects the water column, the whole-plant Q10 would be even higher (4.2), leading to an even more skewed carbon balance with a higher photosynthetic C gain than respiratory C loss.

In conclusion, we found that both respiration and photosynthesis in *Z*. *marina* are significantly affected by plant tissue age, but to different extents. It is therefore important to address age and maturation stage when estimating carbon and oxygen fluxes for seagrasses. The variation patterns in plant metabolism linked to age reported here could have important implications for comparative productivity studies and should be cancelled out by including measurements of a mixture of plant tissue ages. As the Q10 varied substantially depending on the initial temperature level and range chosen, we emphasise that caution needs to be taken when interpreting Q10 results. We also demonstrated that when combining datasets for respiration and photosynthesis, an estimated whole-plant Q10 can be achieved, reflecting the predicted changes in total gas exchange with temperature. In doing so, we showed that the overall increase in the plant’s net O_2_ exchange under realistic maximal temperatures can be three to four times higher than currently estimated. For future studies, we recommend estimating whole-plant productivity at a range of environmentally relevant temperatures, and not just maximal temperatures, to better capture the net O_2_ and CO_2_ fluxes in temperate seagrass beds.

## Materials and Methods

### Study site and plant material

This study was conducted at the Sven Lovén Centre for Marine Sciences (SLC), Kristineberg, Fiskebäckskil, on the Swedish west coast in August 2014. The species studied was the temperate seagrass *Zostera marina*, commonly known as eelgrass. Measurements were conducted at ambient (19 °C) and elevated temperatures (29 °C) on five independent occasions. On each occasion, a seagrass genet (i.e. clonal colony of attached shoots) with at least three shoots (ramets) and the terminal shoot still intact was collected from a seagrass meadow in the vicinity of SLC (58°14′57.40″N, 11°26′48.99″E). The meadow was situated at a depth of approximately 1.5 m. The genets were taken directly to the laboratory, where they were kept in running seawater and exposed to light of ~100 µmol photons m^−2^ s^−1^, i.e. similar to the mean daily irradiance of the meadow (Deyanova *et al*. unpublished data) until the experiment started (from 15 min to 8 h after collection). The youngest shoot of the genet, situated behind the terminal shoot (Fig. 1), was too underdeveloped for the purpose of our study, so the terminal (i.e. oldest shoot) and second youngest shoots were chosen. From each shoot, leaves 2, 3, and 4 were examined. The order of measured shoot age and leaf rank was randomly chosen on each measuring occasion. From each leaf, a 3-cm segment was taken from the base (“basal”), middle (“mid”), and top (“apex”) sections, respectively (Fig. 1). For the determination of respiration rates of the below-ground tissue, a 1-cm segment of rhizome with roots attached was used. For both the terminal and younger shoots, the first node with roots closest to the shoot is the youngest; the second node is of intermediate age and the third node the oldest (Fig. 1). Leaf and rhizome parts were cut using a sharp razor blade.

### Gas exchange measurements

Three incubation chambers (3 mL) connected to Clark type oxygen electrodes (DW1/AD, Hansatech, King’s Lynn, UK) were used to record the oxygen concentration. The oxygen electrodes were controlled through Oxygraph Plus software (Hansatech) and the electrodes were calibrated according to the manufacturer’s instructions before the first measuring round of the day; if unstable results were obtained, the electrodes were recalibrated. Furthermore, the electrodes were recalibrated when new measuring temperatures were used. In each chamber, a seagrass leaf segment was positioned in a U shape to harvest as much light as possible. Saturating light of ~450 µmol photons m^−2^ s^−1^ was provided from the side from a cold light source (KL 1500 LCD, Zeiss, Oberkochen, Germany). Light saturation was determined before the experiment by obtaining rapid light curves using a Diving PAM (Walz, Effeltrich, Germany) (data not shown). In the experiment, natural seawater with oxygen concentrations in equilibrium with air (~100%), a salinity of ~23 (normal for the area), and a pH of 8.1–8.2 was used. The water in the chambers was kept under constant movement using magnetic stirrers. The water temperature was controlled by circulating water of fixed temperatures through jackets surrounding the chambers using a temperature bath (RC20, LAUDA, Lauda-Königshofen, Germany). The temperature was fixed at either 19 ± 0.1 °C (ambient temperature at the collection site) or 29 ± 0.1 °C. During measurements, the experimental set-up was kept in complete darkness for 35 min to obtain steady-state respiration followed by 15 min of light until steady-state photosynthesis was obtained. The root/rhizome measurements were conducted at the end of the experimental period, and oxygen consumption was recorded during 20 min of darkness. The plant material was subsequently dried in an oven at 60 °C for 24 h and weighed to determine the dry weight of the measured samples. The rates of oxygen consumption and evolution (µmol O_2_ gDW s^−1^) were calculated as:

1 $$({\rm{O}}\times 2.6)/1000)/60/{\rm{gDW}}$$

where O is O_2_ (nmol mL^−1^ min^−1^), 2.6 is the fixed water amount in the chamber (ml), /1000 is the conversion from nmol to µmol, /60 is the conversion of min to s, and gDW is the obtained dry weight of sample. Net photosynthesis was corrected for dark respiration rates to obtain apparent photosynthetic rates (sensu^45^). For shoots, rates of all leaf ranks and leaf parts were pooled. Rates of each leaf rank were calculated with the different leaf parts of the rank pooled; rates of each leaf part were calculated with the leaf rank and shoot combined.

Q10 was calculated as:

2 $${({R}_{2}/{R}_{1})}^{10/({\rm{T2}}\mbox{--}{\rm{T1}})}$$

where *R*_2_ is the rate at the higher temperature (*T*_2_) and *R*_1_ is the rate at the lower temperature (*T*_1_). The Q10 value for each tissue part was calculated as a mean of the five replicates at each temperature.

Whole-plant respiratory oxygen consumption (µmol O_2_ gDW^−1^ s^−1^) was calculated as:

3 $$RES{P}_{WP}=({R}_{BG}\times 0.4)+({R}_{AG}\times 0.6)$$

and whole-plant photosynthetic O_2_ evolution (µmol O_2_ gDW^−1^ s^−1^) was calculated as:

4 $$PHO{T}_{WP}=({P}_{BG}\times 0.4)+({P}_{AG}\times 0.6)$$

where *RESP*_WP_ is whole-plant respiration, *R*_BG_ is below-ground tissue respiration, *R*_AG_ is above-ground tissue respiration, *PHOT*_WP_ is whole-plant photosynthetic O_2_ evolution, *P*_BG_ is below-ground tissue photosynthesis, and *P*_AG_ is above-ground tissue photosynthesis. As below-ground tissues are not photosynthetic tissues, oxygen evolution for *P*_BG_ was zero; 0.4 and 0.6 are the values of the biomass ratio between below- and above-ground tissues recalculated from^43,44^.

Whole-plant net O_2_ evolution was calculated as:

5 $$NE{T}_{WP}=RES{P}_{WP}+PHO{T}_{WP}$$

The *NET*_WP_ at 19 °C and 29 °C was used according to Eq. (2) to obtain the whole-plant Q10 (Q10_WP_) for the net O_2_ balance of the plant. For the Q10_WP_, assuming no temperature rise in the sediment, *R*_1_ = *R*_2_ in Eq. (2) for below-ground respiration.

To capture a wider perspective on how the Q10 value may differ depending on the temperature ranges and initial temperatures used, we recalculated data from two previous datasets collected in 2016 and 2017 using the same experimental set-up but with a wider range of temperatures (focusing on above-ground respiration and photosynthesis only). In these studies, only the third youngest leaf from a mixture of shoot ages was used, so the focus was more on exploring the variability of Q10 regarding how the results were calculated. The temperatures used were 10, 15, 20, 25, 30, 35, and 40 °C and the Q10 was calculated for all possible temperature combinations using Eq. (2).

### Statistical analyses

Data on the rates of dark respiration and apparent photosynthesis were analysed and tested using three-factor ANOVAs with Shoot, Leaf, and Part of leaf treated as fixed factors. The significance of differences in the rates of below-ground respiration was tested using two-factor ANOVAs with Shoot and Age treated as fixed factors. Significant main effects (*α* = 0.05) in the ANOVAs were further assessed using Tukey’s post hoc test by comparing pairs of means. Before the ANOVAs, Levene’s^46^ test was performed to check for the homogeneity of variances. Because the criterion of homogeneity was not met, all analyses were performed on log10(*x* + 3)-transformed data.

## Data Availability

All data and results are presented in the manuscript.
